# Reduction of Energy Input in Wire Arc Additive Manufacturing (WAAM) with Gas Metal Arc Welding (GMAW)

**DOI:** 10.3390/ma13112491

**Published:** 2020-05-29

**Authors:** Philipp Henckell, Maximilian Gierth, Yarop Ali, Jan Reimann, Jean Pierre Bergmann

**Affiliations:** Production Technology Group, Technische Universität Ilmenau, D-98693 Ilmenau, Germany; maximilian.gierth@tu-ilmenau.de (M.G.); yarop.ali@tu-ilmenau.de (Y.A.); jan.reimann@tu-ilmenau.de (J.R.); jeanpierre.bergmann@tu-ilmenau.de (J.P.B.)

**Keywords:** additive manufacturing, wire arc additive manufacturing, WAAM, GMAW, energy input per unit length, processing strategy, contact tip to work piece distance, electrical stickout

## Abstract

Wire arc additive manufacturing (WAAM) by gas metal arc welding (GMAW) is a suitable option for the production of large volume metal parts. The main challenge is the high and periodic heat input of the arc on the generated layers, which directly affects geometrical features of the layers such as height and width as well as metallurgical properties such as grain size, solidification or material hardness. Therefore, processing with reduced energy input is necessary. This can be implemented with short arc welding regimes and respectively energy reduced welding processes. A highly efficient strategy for further energy reduction is the adjustment of contact tube to work piece distance (CTWD) during the welding process. Based on the current controlled GMAW process an increase of CTWD leads to a reduction of the welding current due to increased resistivity in the extended electrode and constant voltage of the power source. This study shows the results of systematically adjusted CTWD during WAAM of low-alloyed steel. Thereby, an energy reduction of up to 40% could be implemented leading to an adaptation of geometrical and microstructural features of additively manufactured work pieces.

## 1. Introduction and State of the Art

The industrial application of additive manufacturing processes for metallic parts is growing continuously. A market growth of 41.9% was found for metallic parts in 2018 [1]. This includes technologies such as powder bed fusion (PBF) as well as direct energy deposition (DED) processes. Thereby, a differentiation is made between the applied power source (e.g., laser, electron beam and arc) and the deployed material (e.g., powder and wire) [2]. Herein, differentiations can be adhered in terms of productivity and buildup rates as well as near net shape and surface roughness of manufactured parts.

When using arc-based processes like gas metal arc welding (GMAW), deposition rates of up to 8 kg/h can be achieved [3,4]. Thereby, the buildup volume is only limited by the handling system so that manufacturing of large-volume components can be carried out [4,5]. Though, the dimensional accuracy or surface quality is limited due to comparatively large melt pool sizes. A subtractive post manufacturing process, such as milling is necessary to meet the required tolerances [3,6,7].

In recent years, this technology has been used for the fabrication of high-performance parts in aerospace industry or the energy sector. Therefore, materials such as titanium alloys [8,9] or nickel-base alloys [10,11] have gained increasing interest in the last years. The development of cost efficient production systems [12] with high deposition rates makes this technology accessible for industries such as architecture or construction engineering. Moreover, the freedom of design for complex 3D-structures surpasses the limits of fabrication with conventional methods. Thereby, wire arc additive manufacturing (WAAM) of steel parts is mainly addressed. Recent investigations on low-alloyed steel [13,14,15,16] or high-alloyed steel [17,18] demonstrate the potential for these sectors.

The main challenge in WAAM is the high, periodic heat input due to the arc welding process. Thus, geometrical features of the deposited layers are influenced by large melt pool sizes as well as microstructural properties such as grain size. The analysis and reduction of heat input during WAAM is a key factor for the application of this technology. Therefore, investigations on energy reduced welding regimes were carried out [19,20]. Moreover, cooling strategies were developed to reduce interlayer temperatures and enhance processing times [21,22]. However, the described methods of energy reduction are commonly coupled to specific hardware such as welding power sources with energy reduced processes or additional equipment, e.g., fluid bath, cooling clamps, etc. An applicable approach for the reduction of energy input during WAAM by gas metal arc welding is the extension of the free wire length during processing. Though, electrical properties such as welding current, respectively welding power can be reduced as described below.

**Figure 1:**
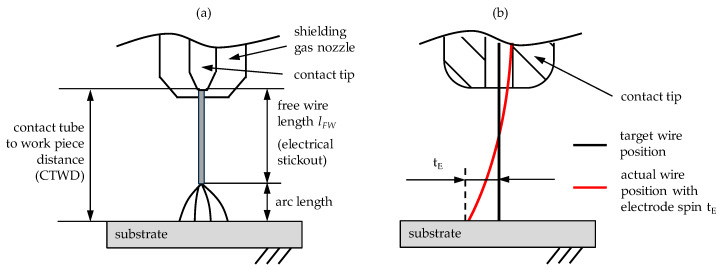
(**a**) Definition of contact tube to work piece distance (CTWD) and (**b**) effect of the electrode spin on wire positioning in gas metal arc welding [26,27,28].

In GMAW, a determining factor for the energy input, the process stability and the formation of the arc is the free wire length *l_FW_*, respectively electrical stickout, which is defined as the distance between the contacting point of the fed wire with the contact tip and the attaching point of the arc on the wire [23,24]. Due to an altering arc length, especially in the short arc welding regime, the electrical stickout is difficult to determine. Therefore, the definition of the contact tube to work piece distance (CTWD) has become established [25,26,27]. In Figure 1a the definition of CTWD and electrical stickout is shown schematically.

The increase of CTWD during the welding process is limited, due to the spin of the electrode resulting from the wire spooling (compare Figure 1b). Subsequently, an offset of the wire tip to the desired welding position *t_E_* may occur with increased CTWD. The consequences are unstable arc behavior, increased spattering or weld seam irregularities such as lack of fusion [29,30]. In WAAM the electrode offset affects the software supported path planning with the risk of dimensional deviations from the CAD model and the work piece [28].

However, the adjustment of the CTWD is a simple and highly efficient strategy to affect energy input in gas metal arc welding, respectively WAAM. This can be demonstrated throughout the following Equations (1)–(4). It has to be stated that a change of CTWD has no influence on the wire feeding speed and almost no influence on the arc voltage [31].

In GMAW, the welding current *I_W_* is conducted over the free wire length *l_FW_* to the arc [32]. With an extended free wire length, the electrical resistance *R_FW_* of the electrode increases [27] (compare Equation (1)). Thereby, *ρ_W_* describes the specific resistance of the wire material and *A_FW_* represents the cross sectional area of the wire. For metallic wires, the specific resistance *ρ_W_* is temperature-dependent and can be described by Equation (2). Thereby, *α* is the temperature coefficient, *T* the temperature and *T*_0_ a temperature at which the specific resistance *ρ_W_ (T*_0_*)* is known, e.g., *T*_0_ = 293.15 K = 20 °C. Equation (2) shows a linear correlation of temperature and specific resistance [33]. Thus, increasing temperature leads to increasing specific resistance for metals and hence affects the electrical resistance of the free wire length *R_FW_*.

(1) $$R_{FW}=\rho_{W}\times \frac{l_{FW}}{A_{FW}}$$

(2) $$\rho_{W}\left(T\right)=\rho_{W}\left(T_{0}\right)\times (1+\alpha \cdot \left(T-T_{0})\right)$$

In addition to the increased resistance of the free wire length *R_FW_*, the set welding current *I_W_* and the resistance of the arc *R_Arc_* influence the welding power *P_W_*. Thereby, the current load of the electrode can be decreased with constantly set welding power (Equation (3)) [24,34]. According to the first Joule’s law, this leads to increased resistance heating of the free wire *Q_FW_* proportional to the square of the welding current *I_W_*, the resistance of the wire *R_FW_* and the welding time *t_W_* (compare Equation (4)).

(3) $$P_{W}=I_{W}^{2}\times \left(R_{FW}+R_{Arc}\right)$$

(4) $$Q_{FW}=I_{W}^{2}\times R_{FW}\times t_{W}$$

As a result, the preheating of the free wire length leads to reduced welding power for melting the electrode. Thereby, the same deposition rate can be achieved [24,27]. A characteristic feature of the resulting material transfer is the formation of larger droplets. This can be described by the softening of the wire throughout the resistance heating as well as a reduction of the electromagnetic pinch-force, due to the reduced welding current. The decreased welding current leads to decreased penetration depth and increased weld seam height in joint welding and cladding [25,32].

The described correlations show a reduction of the welding current at a constant deposition rate, respectively a reduced heat input during the welding process. This makes the process strategy particularly suitable for additive manufacturing using WAAM. So far, the described advantages of CTWD extension have not been investigated in the context of wire arc additive manufacturing and exhibit large potential for the affection of geometrical, microstructural and mechanical properties of additively manufactured work pieces.

## 2. Scope of the Investigations

The objective of this study was the reduction of energy input during WAAM with gas metal arc welding throughout the systematic extension of the contact tube to work piece distance. Thereby, effects on droplet transfer such as resulting droplet diameter or detaching frequency were analyzed by high velocity video recording. Moreover, measurements of welding current and voltage were analyzed to record the resulting reduction of energy input during additive manufacturing. Moreover, investigations on the microstructure and grain size as well as mechanical properties such as material hardness were carried out on low-alloyed steel. Finally, the analysis of cooling rates of the set layers enabled the correlation of interactions between the process strategy and mechanical properties of the built structures.

## 3. Experimental Methods

**Figure 2:**
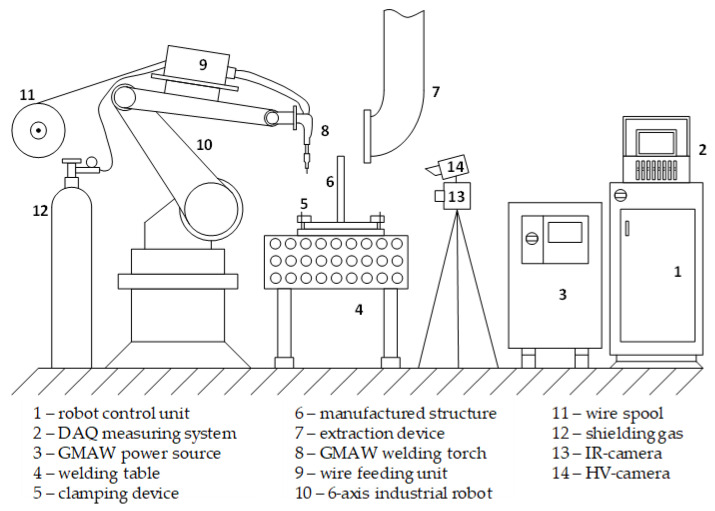
Experimental setup of the wire arc additive manufacturing (WAAM) process.

Additive manufacturing was carried out with a gas metal arc welding process. Therefore, a welding power source of the type Alpha Q 552 Puls (EWM AG, Mündersbach, Germany) and a water-cooled welding torch WH W 500 (Alexander Binzel Schweißtechnik GmbH & Co. KG, Buseck, Germany) was used. The reproducible guidance of the welding torch was realized by a 6-axis industrial robot KR15-2 (KUKA AG, Augsburg, Germany). The GMAW process was carried out in short arc welding regime throughout the present case study. During WAAM, a temperature controlled strategy with flexible dwell time was implemented. Therefore, the welded layers were naturally cooling to 100 °C before the next layer was applied. The process was monitored with IR-camera of the type ImageR 8320 (InfraTec GmbH, Dresden, Germany). This strategy prevents excessive heat input and heat accumulation in the structure and enables comparable cooling conditions for the analysis of microstructural evolution. Therefore, cooling rates were simultaneously recorded by IR-camera in the range between 900 and 400 °C with a frequency of 50 Hz. The camera was positioned horizontally to the manufactured wall structures with a distance of 600 mm. The analysis of cooling rates was carried out with the software IRBIS 3 plus (InfraTec GmbH, Dresden, Germany). The experimental setup is shown in Figure 2.

High velocity camera imaging was carried out with HV-camera of the type CR2000x2 (Optronis GmbH, Kehl, Germany) to analyze arc behavior and droplet transfer during GMAW. The frame rate was set to 2500 fps at a resolution of 800 pixels × 600 pixels. The shutter speed was set to 1/10.000 s and the aperture adjusted manually to fade-out the arc partially in order to visualize droplet transfer.

Measurement data was recorded by a Dewetron DEWE-PCI 16 measuring system (version DEWE-800, Dewetron GmbH, Grambach, Austria) with data acquisition of current, voltage and welding power. The generated wall structures were separated from the substrate by wire-electro discharge machining (EDM). Afterwards the samples were prepared for metallographic analysis including grinding, polishing and etching with alcoholic Nital solution based on 3% nitric acid. The hardness measurements were carried out using a DuraScan 70 machine (Struers GmbH, Willich, Germany) using the Vickers testing method according to [35] with a force of 9.807 N (HV1).

Low-alloyed steel S355 J2 + N (1.0570) was used as substrate material with a thickness of 20 mm. A solid wire of low-alloyed steel G4Si1/SG3 (1.5130, Ø = 1.0 mm) was used as filler material in the GMAW process. Herein, the silicon percentage was increased by 0.3% by the manufacturer to enhance the binding of oxygen and reduce the formation of porosity in the weld bead. Prospectively, the chosen filler material is of high interest for industrial sectors such as construction engineering and architecture. The following Table 1 shows the chemical composition of the substrate material and filler wire for WAAM. During additive manufacturing, 98%Ar/2%CO_2_ was used as shielding gas for the GMAW process.

**Figure 3:**
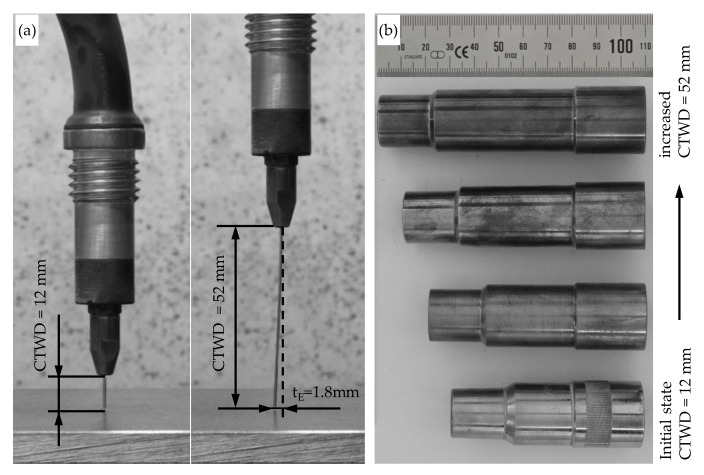
(**a**) Measurement of electrode spin with increased CTWD and (**b**) adaptation of shielding gas nozzles for gas metal arc welding (GMAW) with increased CTWD.

During experimental trials, the contact tube to work piece distance was systematically increased in steps of 8 mm. The starting value was set to CTWD = 12 mm, which is the standard distance based on the formula CTWD = 10…12 × Ø_wire_. The maximum extension was set to CTWD = 52 mm. Referring to the state of the art, a spin of the welding electrode *t_E_* could be observed resulting from the wire spooling. The maximum measured value of *t_E_* is shown in Figure 3a at the maximum extension of CTWD = 52 mm. Hereby, the electrode spin was measured at a constant value of *t_E_* = 1.8 mm. A commonly displaced position of the electrode to one direction does not affect the additive manufacturing process and can be compensated throughout the programming of an offset in the handling system.

In order to provide constant gas shielding conditions on the weld bead, a constructional adaptation of the shielding gas nozzles was applied for the experimental trials with increased CTWD. Herein, the increased distance between substrate and contact tube requires a focused gas flow to the weld bead equally to the initial setup. Figure 3b shows the adjustment of the lengths of the shielding gas nozzles for increased CTWD.

## 4. Results and Discussion

### 4.1. Analysis of Elctrical Properties in WAAM with Increased CTWD

**Figure 4:**
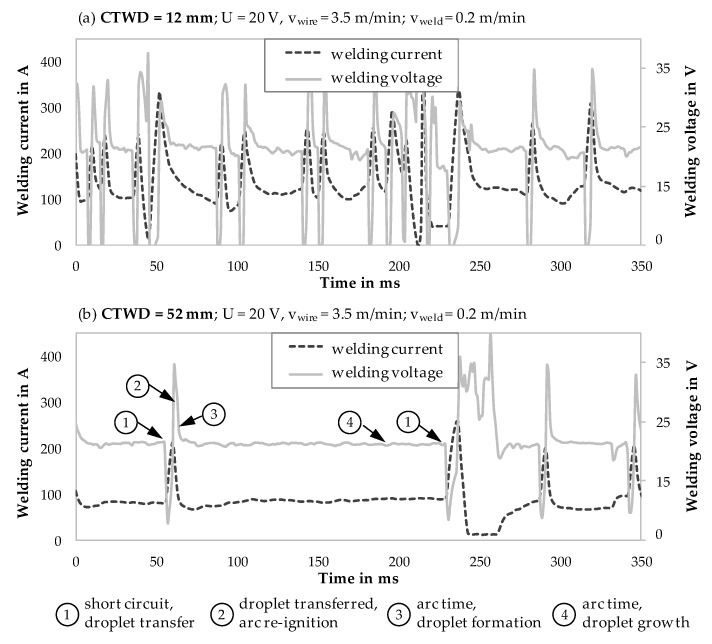
Analysis of welding current and voltage in GMAW with varied contact tube to work piece distance (CTWD); (**a**) CTWD = 12 mm and (**b**) CTWD = 52 mm.

In the first step, electrical properties were analyzed during WAAM with a short arc welding regime to characterize the stability of the arc and short arc frequency. Welding voltage and current were measured with a rate of 1000 Hz for GMAW processes with varied CTWD from 12 to 52 mm. The following Figure 4 shows welding current and voltage for a period of 350 ms (Figure 4a CTWD = 12 mm; Figure 4b CTWD = 52 mm). Furthermore, characteristic points of the electrical signals and droplet formation in short arc welding regime are exemplarily shown in Figure 4b throughout circled numbers in the diagram.

Short circuits are characterized by deflections in welding current and voltage in relation to the base values. Herein, a rapid voltage drop with increasing current could be recognized (compare Figure 4b, circle number 1). At this point, the molten droplet transferred from the end of the electrode into the melt bead. A material bridge between the fed wire and the melt pool occurred. After material transition the arc re-ignited. Therefore, high voltage was required, which was characterized by peaks in welding voltage (compare Figure 4b, circle number 2). At this point, welding current dropped slowly due to inductances in the welding circuit, leading to high welding power during arc re-ignition. The subsequent period of arc time led to the melting of the electrode tip and the formation of a new droplet (compare Figure 4b; circle number 3). Thereby, increasing arc time promoted droplet growth (compare Figure 4b; circle number 4) before the molten material transferred into the melt bead during the next short circuit phase.

Figure 4 shows differences in short circuit frequency and uniformity of the short circuits over time with increasing CTWD. Thereby, Figure 4a shows homogeneous droplet transfer with periodical short circuits of f = 42 Hz with a CTWD of 12 mm. Increasing CTWD leads to a reduction of the short circuit frequency (compare Figure 4b). This can be explained by the formation of large volume droplets due to decreased welding current respectively decreased pinch force. Thus, Figure 4b shows a short circuit frequency of f = 11 Hz with increased CTWD of 52 mm. The irregularity of the short circuit frequency arose through the formation of larger droplets (compare Figure 4b; circle number 4), which were transferred with higher kinetic energy into the melt pool causing a wave formation of the liquid melt pool. These waves partially reached the newly forming drop at the end of the wire, creating a new short circuit. This effect caused the transfer of smaller droplets with a reduced detaching frequency during short circuit. The described effect is shown in Figure 4b in the time window between 200 and 350 ms.

It can be shown that increased CTWD led to a reduction of droplet transfers in the short arc welding regime. Concurrently, phases of arc re-ignition with high welding power decline, leading to energy reduction induced by the welding regime and respectively the arc. As a result, the energy input per unit length *E_s_*, as a main dimension for the characterization of arc welding processes, can be reduced with a constantly set welding velocity. Equation (5) describes the correlation between welding power *P_weld_* and welding velocity *v_weld_* Herein, welding voltage *U_weld_* and current *I_weld_* can be measured directly to estimate welding power *P_weld_* (compare Equation (6)).

(5) $$E_{S}\left[\frac{\mathrm{kJ}}{\mathrm{cm}}\right]=\frac{P_{weld}}{v_{weld}}\left[\frac{\mathrm{kW}}{\frac{\mathrm{cm}}{s}}\right]$$

(6) $$P_{weld}=\frac{1}{t}\overset{t}{\underset{0}{\int }}p_{weld}\left(t\right)dt=\frac{1}{t}\overset{t}{\underset{0}{\int }}u_{weld}\left(t\right)i_{weld}\left(t\right)dt$$

**Figure 5:**
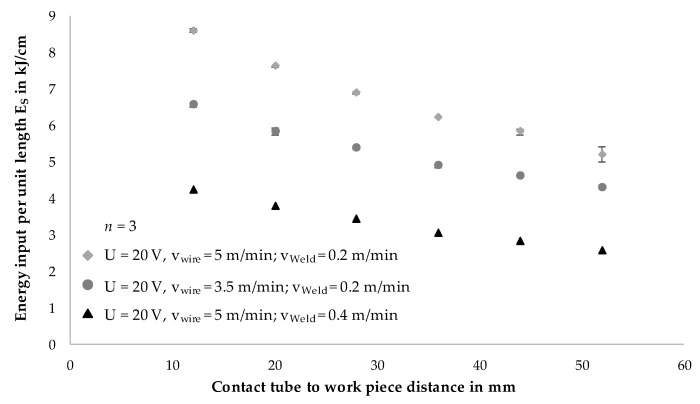
Analysis of energy input per unit length with increasing CTWD for differing parameter sets.

The following Figure 5 shows the correlation of energy input per unit length *E_S_* to the contact tube to work piece distance (CTWD) for three parameter sets. Thereby, *E_S_* was set to values between 4.2 and 8.2 kJ/cm in the initial state of 12 mm CTWD. Increasing CTWD from 12 to 52 mm shows a steady reduction of the energy input per unit length. This relation is represented independently from the parameter set. It was evident that an increase in CTWD of 8 mm led to a reduction of energy input per unit length of 8%. A CTWD of 52 mm exhibited the maximum reduction in energy input per unit length of 40% compared to the initial state. These results correspond to the state of the art in the field of joint welding and cladding as described [25,31].

### 4.2. Analysis of Droplet Transfer in WAAM with Increased CTWD

**Figure 6:**
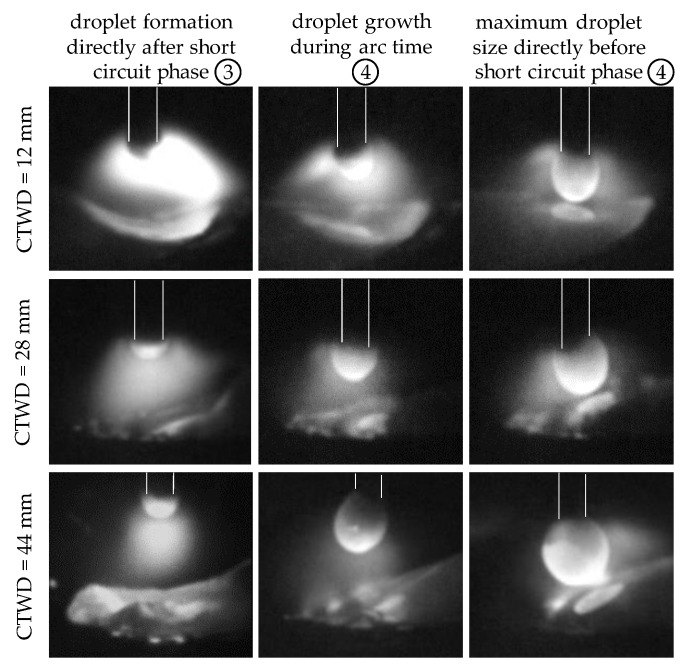
Droplet formation during GMAW with increasing CTWD for U = 20 V; v_wire_ = 5 m/min and v_weld_ = 0.4 m/min.

Chapter 4.1 verified alterations in short circuit frequency with adapted CTWD throughout the analysis of electrical properties. Referring to the state of the art, increasing CTWD leads to the formation of large volume droplets during material transfer in GMAW [25,32]. Experimental investigations with a high velocity video recording confirmed a constant droplet growth with increasing CTWD. Figure 6 shows droplet formation in GMAW with differing CTWD of 12 mm, 28 mm and 44 mm at characteristic points during the short arc welding regime and correlated to the circled numbers in Figure 4b. The increase of CTWD is shown in uniform step size of 16 mm for equal welding parameters. Thereby, the formation of a new droplet on the electrode directly after short circuit phase is shown (compare Figure 4b, circle number 3) as well as droplet growth during arc time (compare Figure 4b, circle number 4). Furthermore, the maximum droplet size is displayed directly before the material transfer in the short circuit phase. The focus of the investigations was set to analyze droplet growth and size before material transition into the melt bead. Therefore, the short circuit phase with material transfer (compare Figure 4b, circle number 1) and arc re-ignition (compare Figure 4b, circle number 2) is not shown in Figure 6.

**Figure 7:**
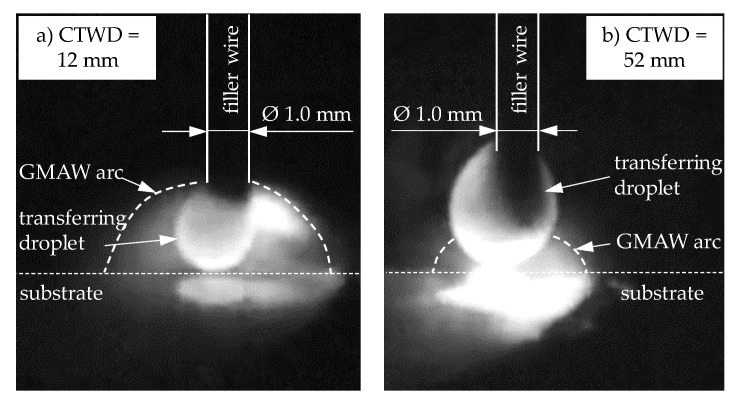
Correlation of droplet growth and arc behavior in GMAW with increased CTWD for U = 20 V; v_wire_ = 5 m/min and v_weld_ = 0.4 m/min.

The coupled process development of increasing droplet size and reduced short circuit frequency was caused by increasing resistance heating of the free wire length with increasing CTWD. Due to high temperatures, the extended wire softened. This effect enhanced the formation of droplets with larger material volume at the end of the wire. At the same time, the pinch effect was reduced, which was induced by the Lorentz force of the magnetic field. The reason for this is the lower welding current that affects the Lorentz force squared. Figure 7a shows droplet formation for CTWD of 12 mm and the maximum investigated value of 52 mm (Figure 7b) in detail. Herein, characteristic effects in the formation of the arc and the resulting droplet can be shown with differing CTWD.

Thereby, a contact tube to work piece distance of 12 mm indicates the arc attaching point at the filler wire, enclosing the formed droplet at the end of the electrode (compare Figure 7a). At this point, the electromagnetic pinch force was applied, constricting the molten droplet. However, increasing CTWD led to a displacement of the arc attaching point to the bottom of the formed droplet (compare Figure 7b). This resulted in a reduced pinch force to constrict the molten material, due to a decreased welding current. The formation of large volume droplets was the consequence.

**Figure 8:**
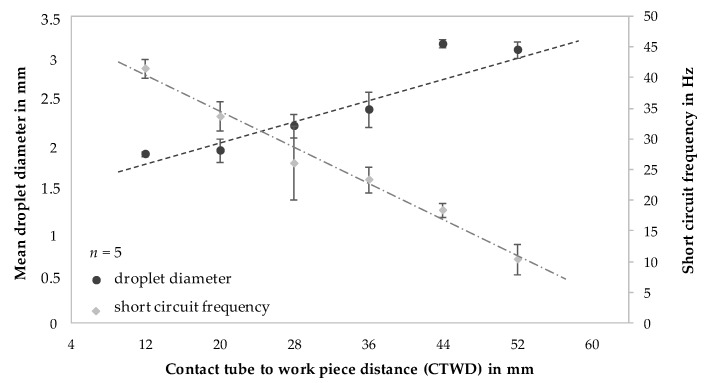
Correlation of mean droplet diameter and short circuit frequency with differing CTWD for U = 20 V; v_wire_ = 5 m/min and v_weld_ = 0.4 m/min.

The described correlation between short circuit frequency (compare Figure 4) and droplet volume (compare Figure 7) with systematically varied CTWD is shown in Figure 8. To determine the mean droplet size, high-speed camera recordings were analyzed and droplet diameters measured shortly before the transition into the melt pool on five consecutive droplets. The wire width served as a reference for the measurements in the 2-dimensional images. The short circuit frequency was determined on the basis of the number of current peaks, respectively voltage drops for a period of one second during the welding process. In the short arc welding regime the number of short circuits equaled the number of droplet transfers into the melt pool.

Apparently, the short circuit frequency decreased with increasing CTWD. In the initial state of CTWD = 12 mm the short circuits occurred with a mean frequency of 42 Hz. This frequency was reduced to 11 Hz with increased distance between contact tube and substrate of 52 mm. This corresponded to 74% of the initial value. At the same time, droplet diameter increased throughout the previously described effects. The mean droplet size was determined to be 1.9 mm at 12 mm CTWD. The increase of the distance from contact tube to substrate up to 52 mm resulted in increased droplet sizes with a mean value of 3.1 mm. Thus, the average droplet size had increased by approximately 64%. During the experimental investigations, a maximum CTWD of 52 mm led to an inhomogeneous process behavior with arc deflections and excessive spatter formation. Further experimental trials were carried out with a maximum value of CTWD = 44 mm.

**Figure 9:**
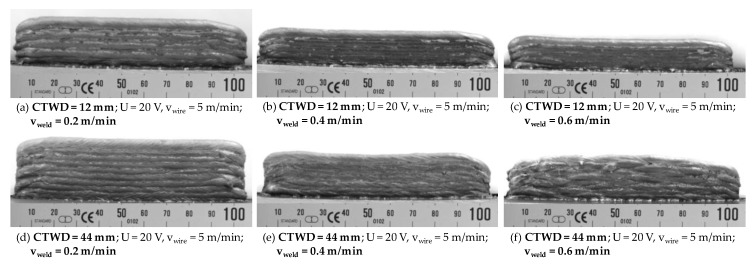
Correlation of CTWD adjustment and differing welding speeds on wetting behavior and near net shape quality during WAAM.

A side effect of decreasing short circuit frequency and respectively material transfer with large volume droplets affected the wetting behavior during GMAW. Herein, the formation of large volume droplets with low transition frequency led to a punctual wetting of the substrate. In context of WAAM, the wetting behavior was coupled to the welding velocity and was significantly relevant for the near net shape quality of the built structures. A homogeneous material transfer resulted in the formation of layers with equal geometrical properties such as width and height. Figure 9 shows the influence of CTWD adjustment and differing welding velocities on the geometrical formation of layers during WAAM. Thereby, a CTWD of 12 mm, respectively 44 mm was adjusted with differing welding velocities of 0.2–0.6 m/min. Wall structures of 100 mm length and 10 layers were manufactured. The temperature controlled WAAM process was executed with alternating welding direction in each layer and constantly set interlayer temperature of 100 °C before the next layer was applied.

It becomes apparent, that surface quality and structure height of the work pieces were affected by CTWD adjustment as well as welding velocity. Systematical investigations on geometrical properties during WAAM with extended CTWD are shown in Chapter 4.3.

Figure 9a–c exhibited near net shaped quality for CTWD adjustment of 12 mm and welding velocities from 0.2 to 0.6 m/min. Thereby, little curvature at the structure beginning and end, an even overall height and low spatter formation were observed. In addition, high frequency of short circuits (compare Figure 8) led to homogeneous wetting behavior and melt bead solidification. As a result, the required amount of post-processing was limited due to geometrical properties close to the final contour. Increasing welding velocity results in the formation of structures with decreased height, which could be explained by reduced material transfer per unit length at a constant wire feeding speed.

Furthermore, WAAM with increased CTWD of 44 mm and adapted welding velocity is shown in Figure 9d–f. Thereby, the reduced short circuit frequency (compare Figure 8) affected the wetting behavior and near net shape properties. Thus, a stable welding process and the formation of layers with equal geometrical properties could be achieved with low welding velocities up to 0.4 m/min (compare Figure 9d,e). Thereby, surface roughness as well as the curvature at the structure beginning were slightly affected. As a result, requirements for near net shape quality in WAAM could be met with an increased CTWD of 44 mm.

Negative affection of process behavior and near net shape quality with increased CTWD is shown in Figure 9f. Herein, the combination of the reduced frequency of droplet transfers and increased welding velocity of 0.6 m/min led to partial wetting of the previously set layers. This resulted in inhomogeneous layer geometries during the manufacturing process. Throughout these dimensional deviations in layer height, the arc ignition after the short circuit phase as well as the arc length was affected constantly. The unstable welding process was insufficient for WAAM processing. As a result, the described properties for near net shape quality could not be met.

### 4.3. Effects of Increased CTWD on Geometrical Layer Properties in WAAM

**Figure 10:**
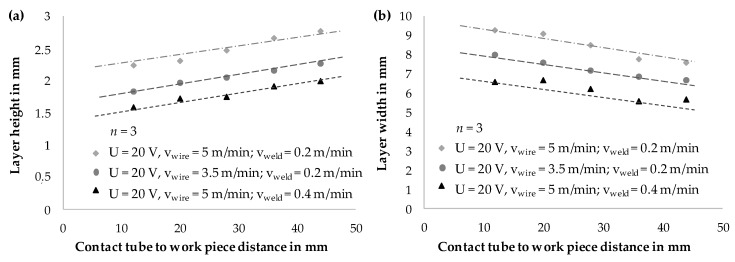
(**a**) Determination of layer height and (**b**) analysis of layer width for 3 parameter sets and adjusted CTWD.

In gas metal arc welding, increasing CTWD led to the reduction of energy input per unit length *E_S_* respectively heat input as described (compare Figure 5). This had a direct effect on the formation of the weld bead geometry at a constant deposition rate. Due to lower energy input, the weld seams increased in height and decreased in width continuously. This could be described by lower temperatures in the melt pool and thus a lower viscosity of the melt, resulting in less flowable melt and faster solidification of the molten material. Figure 10 shows experimental results for the adaptation of CTWD from 12 to 44 mm in the context of resulting layer geometries. Three parameter sets with differing energy input per unit length were chosen. Though, layer height (compare Figure 10a) and layer width (compare Figure 10b) were measured on three positions of a wall structure with 10 layers. The mean values are shown in Figure 10.

The diagram in Figure 10a shows increasing values for layer height with increasing CTWD. A rise of approximately 25% was observed independently from the parameter set. The examination of the layer width (compare Figure 10b) exhibited an inverse dependence. Herein, layer width decreased to approximately 83% (CTWD = 44 mm) of the initial value (CTWD = 12 mm).

**Figure 11:**
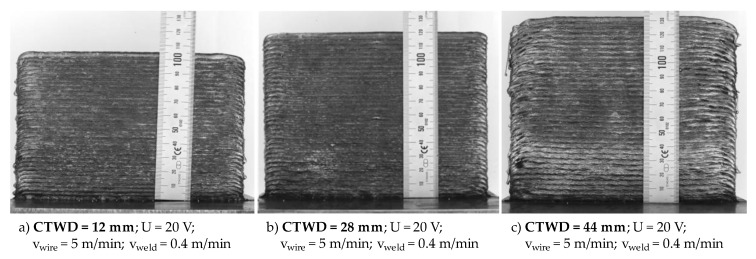
WAAM of wall structures with 70 layers and differing CTWD.

In the next step of the investigations, wall structures with a length of 160 mm and 70 layers were manufactured to analyze process behavior and near net shape quality on large volume work pieces. Figure 11 exemplarily shows the resulting work pieces for a CTWD of 12 mm (compare Figure 11a), 28 mm (compare Figure 11b) and 44 mm (compare Figure 11c). On these structures, microstructure and material hardness were tested additionally (compare Chapter 4.4). Therefore, the applied parameters were economically set to reduce processing time. The experimental trials were carried out with a welding velocity of 0.4 m/min, a voltage of 20 V and a wire feed of 5 m/min. The welding current as well as the energy input per unit length depend on the adjusted CTWD and varied from 4.23 (CTWD = 12 mm) to 2.82 kJ/cm (CTWD = 44 mm). The increase in CTWD from 12 to 44 mm led to a reduction of energy input per unit length of 34% (compare Figure 5). The interlayer temperature was set to 100 °C between the deposited layers.

Figure 11 shows the resulting wall structures consisting of 70 layers. Apparently, the structures increase in height with extended CTWD as described before (compare Figure 9). At the same time, surface roughness increased due to the described droplet diameter, transferring frequency and wetting properties. The resulting mean structure height and mean layer height are shown in Table 2. Herein, the values for mean structure height were measured on three points of the wall structures and the values for mean layer height calculated by the division of mean structure height by the total number of layers. Furthermore, the results were compared to preliminary manufactured wall structures with 10 layers (compare Figure 10). The deviation is also shown in Table 2. A CTWD of 12 mm results in a structure height of approximately 107 mm, respectively a mean layer height of 1.53 mm (compare Table 2). Increased CTWD of 44 mm led to a structure height of approximately 134 mm (1.92 mm layer height), which was an increase of 25%. Little deviations < 5% compared to structures in preliminary investigations confirmed process stability and the effects of increasing CTWD on geometrical layer properties on large-volume structures.

The side effect of adapted layer geometries was significant for the use of increased CTWD during WAAM. The change of the weld bead geometry at constant welding voltage, constant welding speed and constant wire feed has to be taken into account for path planning strategies. Especially if the value of the contact tube to work piece distance is set dynamically in the WAAM process to adapt the layer height or width.

### 4.4. Effects of Increased CTWD on Microstructure Development in WAAM

**Figure 12:**
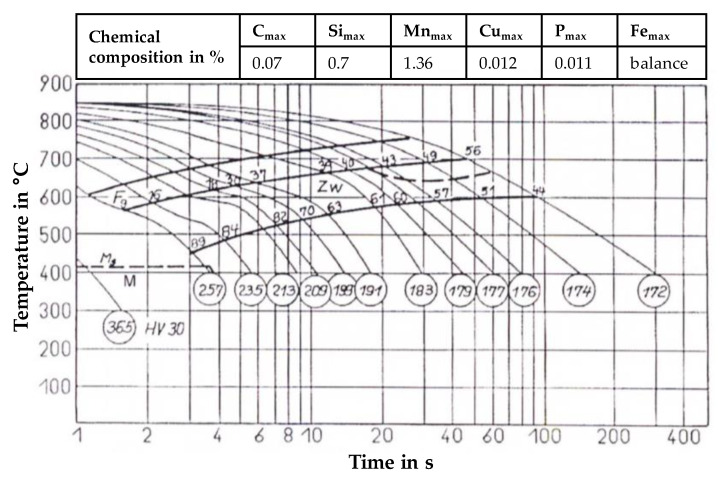
CCT-diagram of low-alloyed steel SG36 with comparable chemical composition to the used welding wire G4Si1/SG3 (1.5130) [37].

In wire arc additive manufacturing, the adjustment of energy input affects the cooling conditions and moreover metallurgical properties of the metallic work piece. Though, microstructure and mechanical properties can be influenced. A common approach to characterize un- and low-alloyed steel during welding is the estimation of the t_8/5_-time according to DIN EN 1011-2:2001-05 [36]. This describes the time interval of cooling of the weld bead and the heat-affected zone (HAZ) from 800 to 500 °C. In this temperature range, phase transformations from γ- to α-phase take place, which are decisive for the mechanical-technological properties. Therefore, a continuous cooling transformation (CCT) diagram can be used to estimate microstructure and mechanical properties in dependence on the chemical composition and occurring cooling rates. Since no diagram of the applied filler material is available from the manufacturer, a CCT-diagram of steel with a chemical composition as similar as possible was used. Figure 12 shows the CCT-diagram of the low-alloyed steel SG36 as well as the chemical composition [37]. Furthermore, phase formation at different cooling rates and expectable hardness values are shown. Though, minor deviations in silicon and manganese content of approximately 0.3% can be seen. Due to this, slight deviations in phase proportions and hardness in comparison to the used filler wire G4Si1/SG3 may occur.

The filler material is a hypo-eutectoid steel with a low carbon content of C ≤ 0.07% and increased percentage of silicon and manganese. Thus, almost no pearlite was formed during cooling (compare Figure 12). The microstructure was mainly composed of different proportions of intermediate structure (bainite) and ferrite at moderate cooling rates. At very high cooling rates, e.g., in the first weld bead, martensite formed.

**Figure 13:**
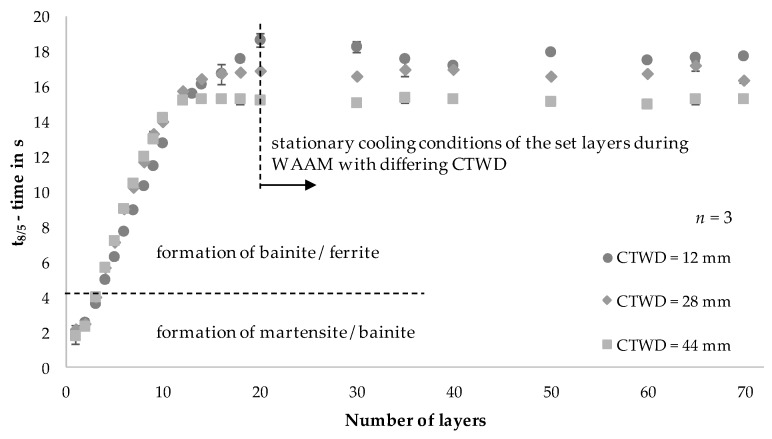
Analysis of the t_8/5_-time in dependence on the number of layers and CTWD for U = 20 V; v_wire_ = 5 m/min and v_weld_ = 0.4 m/min (mean values of 3 measuring points per layer).

To correlate process adaptations and resulting metallurgical properties of the previously built structures (compare Figure 11), cooling rates of the set layers were analyzed during WAAM with adapted CTWD. Therefore, t_8/5_-times were recorded layer wise by IR imaging. The analysis was carried out on each of the first 10 layers due to strongly varying cooling rates. This can be explained by heat conduction to the substrate, which featured room temperature in the initial state. As a result, an increasing number of layers led to heat accumulation and rising temperatures in the substrate until a quasi-stationary condition of heat input and heat transfer was reached. During the manufacturing of the following 10 layers, measurements were carried out in every second layer. Finally, every 10th layer was recorded with the exception of layer 35, which is the center of the structure. Figure 13 shows the resulting t_8/5_-times during WAAM depending on the layer height and the set CTWD.

The measured t_8/5_-times in the 1st weld bead of approximately 2 s show small deviations between the experimental trials with adjusted CTWD. According to the CCT-diagram, the formation of a martensitic-bainitic microstructure with high hardness values > 257 HV appeared for t_8/5_-times up to 4 s. These cooling conditions were measured from the first to the third layer (compare Figure 12). With increasing structure height and thus longer t_8/5_-times, the microstructure consisted of varying proportions of bainite and ferrite (compare Figure 13). The expectable hardness decreased.

Between the 4th and 10th layer, the t_8/5_-times for a CTWD of 12 mm were slightly lower of approximately t_8/5_ = 1.5 s in comparison to a CTWD of 28 mm, respectively 44 mm. This development is controversial to the process behavior and reduced energy input per unit length with increasing CTWD. The reason for the opposite development is the fact that the weld bead geometry and thus the conditions of heat conduction to the substrate change with increasing contact tube distance. The width of the weld bead (compare Figure 10b) decreased by approximately 0.6 mm (CTWD = 28 mm) or 0.9 mm (CTWD = 44 mm) and consequently also the effective area for heat conduction. The effect of thermal conduction is the essential driver of heat transfer close to the substrate. This observation is consistent with the research of Cunningham et al. [7].

From the 12th layer on, the resulting t_8/5_-times adjusted to an increasingly stationary state between 15 and 20 s. This can be explained by the low amount of introduced thermal energy and the resulting lower temperature gradients between the structure and the substrate. As a result, stationary cooling conditions of the set layers can be observed. Herein, a difference in the t_8/5_-time of approximately 2.5 s results for the different CTWD adjustments. A correlation between the energy input per unit length (compare Figure 5) and the resulting cooling rates (compare Figure 13) can be shown throughout decreasing t_8/5_-times at reduced energy input. According to the CCT-diagram [37], a microstructural composition of 63% bainite and 37% ferrite occurred for t_8/5_-times between 15 to 20 s with hardness values below 191 HV.

Table 3 shows the estimated phase formation and hardness values for the 1st, 35th and 70th layer. It should be noted that a CCT-diagram [37], which is designed for a chemically comparable steel was used. Due to this, slight deviations of the theoretical values to the experimentally measured values have to be taken into account.

**Figure 14:**
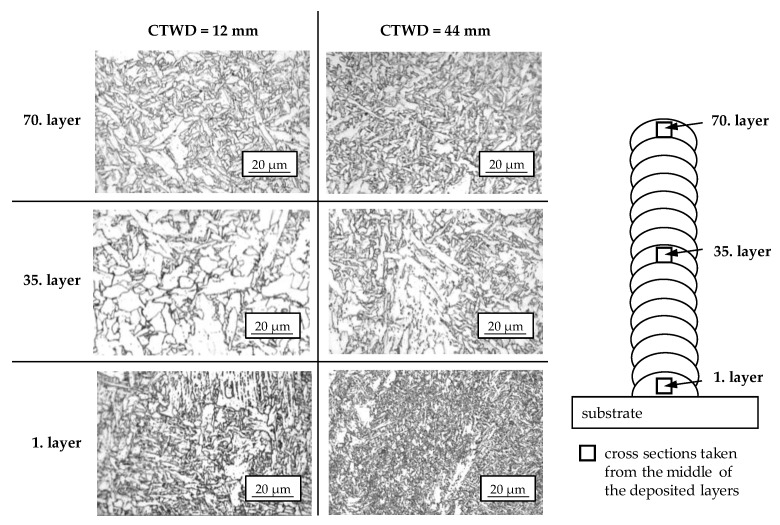
Microstructural cross sections of additively manufactured walls with differing CTWD for U = 20 V; v_wire_ = 5 m/min and v_weld_ = 0.4 m/min.

In the next step, microstructural cross sections of the previously built wall structures with varied CTWD of 12 mm and 44 mm were taken from the middle of the 1st, 35th and 70th layer. Herein, microstructural evolution of low-alloyed steel G4Si1/SG3 is shown in dependence on differing cooling conditions during WAAM. The cross sections are shown in Figure 14.

In the cross sections of the 1st layer, a primarily bainitic structure with accumulations of ferrite grains is apparent for a CTWD of 12 mm and 44 mm. According to Seyffarth et al. [37], martensitic phase can be seen in addition to the bainitic microstructure due to t_8/5_-times < 2 s in the first layer. With increasing CTWD of 44 mm, the microstructure becomes fine grained, although the measured cooling rates were equal. This phenomenon can be explained by lower energy input with increasing CTWD (compare Figure 5). Thereby, lower heat input resulted in decreased grain growth as well as decreased dilution between filler material and substrate.

The cross sections of the 35th layer show a microstructural composition of coarse-grained ferrite with embedded globular bainite for a CTWD of 12 mm. An increased CTWD of 44 mm led to a reduced grain size. This correlated to the decreased energy input per unit length (compare Figure 5) and reduced t_8/5_-times in the center of the structure (compare Figure 13). Furthermore, a periodical reheating of the bead throughout the following layers enhanced grain growth. In the 70th layer a bainitic-ferritic structure is visible, independently from the adjusted CTWD. This corresponds to the state of the art [37]. The fine grained microstructure can be explained by a lack of post-weld heat treatment in the last layer of the WAAM process. Further heat input throughout a following layer is not present.

Throughout microstructural testing, no structural defects such as a lack of fusion (compare Figure 11) or porosity could be found. Herein, low energy input per unit length (compare Figure 5) led to the formation of small and stable melt beads with enhanced decarburization. Furthermore, the increased silicon content of 1.0% (compare Table 1) in the filler wire provided enhanced binding of oxygen during solidification.

**Figure 15:**
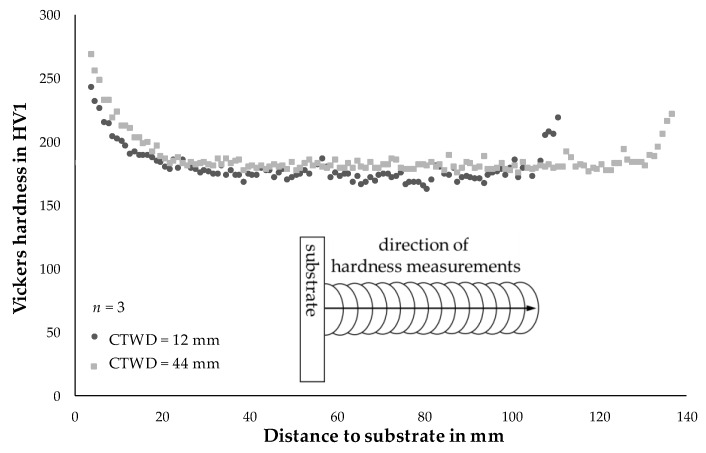
Hardness measurements of WAAM structures with differing CTWD for U = 20 V; v_wire_ = 5 m/min and v_weld_ = 0.4 m/min.

The verification of microstructural phases was carried out by hardness measurements on the wall structures described in Chapter 4.3. Therefore, Vickers hardness testing was conducted from the substrate to the top layer in build-up direction of the work pieces. Figure 15 shows the resulting mean values of three parallel measuring rows per structure for Vickers hardness testing with a distance of 1 mm between the measuring points.

A CTWD of 44 mm led to mean hardness values of 271 HV1 in the 1st layer, which was approximately 30 HV1 higher than the mean hardness value at 12 mm CTWD in the same position. According to the CCT-diagram of the unalloyed steel, martensitic phase exceeds hardness values of 257 HV1 [37] and can be shown for a CTWD of 44 mm. Despite equal t_8/5_-times between 12 and 44 mm CTWD, this can be explained by reduced heat input and less dilution between filler material and substrate. As a result, a fine grained microstructure (CTWD = 44 mm) led to increased hardness. Moreover, the slightly different chemical composition of the filler wire to the used CCT-diagram must be considered. Increasing percentage of silicon and manganese of approximately 0.3% might affect martensite start temperature as well as phase fraction.

With increasing number of layers, the difference in hardness becomes smaller. From a distance to the substrate of approximately 20 mm a mean hardness of 180 HV1 can be observed for a CTWD of 44 mm. A decreased CTWD of 12 mm led to mean hardness values of approximately 168 HV1. These values can be measured with little deviation throughout the additively manufactured structure to a distance from the substrate of 102 mm (CTWD = 12 mm) respectively 126 mm (CTWD = 44 mm). The values correspond to the shown cross sections of the 35th layer (compare Figure 12) and verify the presence of ferritic phase with proportions of bainite according to the CCT-diagram [37].

Independently from the set CTWD an increase in hardness can be seen in the last layers of the structures up to 220 HV1 (compare Figure 15). This refers to a lack of post-heat treatment in the last layers and a fine-grained microstructure.

## 5. Summary and Outlook

In this study the influence of the contact tube to work piece distance (CTWD) was investigated in the context of energy reduction in wire arc additive manufacturing (WAAM). Investigations on electrical properties such as energy input per unit length *E_S_* or short circuit frequency were analyzed. It could be shown that an increase in CTWD of 10 mm led to a reduction in *E_S_* of 10%. A maximum reduction of 40% could be achieved. Thereby, droplet detaching frequency was reduced throughout decreasing welding current respectively reduced pinch force. As a result, the formation of increasing droplet sizes was observed. However, the adaptation of CTWD affected geometrical properties of the deposited layers as well as microstructural properties of the investigated steel. It was shown, that the grain size could be influenced by the adjustment of CTWD due to differing cooling rates. As a result, modified material hardness in the structure emerges.

In WAAM, the adaptation of CTWD is a simple and highly efficient approach, which can be applied as inline processing strategy in order to regulate energy input during the build-up process. Furthermore, the processing strategy can be used for wire arc additive manufacturing of work pieces with tailored properties within the built structures. Herein, dimensional and microstructural modulations can be implemented.

## Figures and Tables

**Table 1:** Chemical composition of substrate and welding wire (%).

Function	Material	C_max_	Si_max_	Mn_max_	Cu_max_	Fe_max_
substrate	S355J2 + N (1.0570)	0.20	0.55	1.60	0.55	balance
welding wire	G4Si1/SG3 (1.5130)	0.07	1.00	1.64	0.05	balance

**Table 2:** Structure height of large WAAM structures and comparison to previous trials (compare Figure 10).

CTWD (mm)	Mean Structure Height (mm)	Mean Layer Height (mm)	Deviation (%)
12	106.8 ± 0.3	1.53	−4.5
28	121.7 ± 0.2	1.74	−1.2
44	134.1 ± 1.4	1.92	−4.2

**Table 3:** Estimation of phase composition and hardness depending on the t_8/5_-time according to the CCT-diagram [37] (compare Figure 12).

CTWD (mm)	Number of Layer	t_8/5_-Time (s)	Phase Formation (%)	Hardness (HV30)
12; 28; 44	1	1.8–1.9	Martensitic-bainitic	>257
	35	15.3–17.5	63% bainite, 37% ferrite	<191
	70	15.4–17.7	63% bainite, 37% ferrite	<191

## References

[1] Wohlers Report 2019 Details Striking Range of Developments in AM Worldwide. https://www.3dprintingmedia.network/wohlers-report-2019-details-striking-range-of-developments-in-am-worldwide/.

[2] Karunakaran K.P., Bernard A., Suryakumar L.D., Taillandier G. (2012). Rapid manufacturing of metallic objects. Rapid Prototyp. J..

[3] Karunakaran K.P., Suryakumar S., Pushpa V., Akula S. (2010). Low cost integration of additive and subtractive processes for hybrid layered manufacturing. Rob. Comput. Integr. Manuf..

[4] Wu B., Pan Z., Ding D., Cuiuri D., Li H., Xu J., Norrish J. (2018). A review of the wire arc additive manufacturing of metals: Properties, defects and quality improvement. J. Manuf. Process..

[5] Candel-Ruiz A., Kaufmann S., Müllerschön O. (2015). Strategies for high deposition rate additive manufacturing by laser metal deposition. Proceedings of Lasers in Manufacturing (LiM).

[6] Bergmann J.P., Henckell P., Reimann J., Ali Y., Hildebrand J. (2018). Grundlegende wissenschaftliche Konzepterstellung zu bestehenden Herausforderungen und Perspektiven für die Additive Fertigung mit Lichtbogen. Weld. Cut.

[7] Cunningham C.R., Flynn J.M., Shokrani A., Dhokia V. (2018). Invited review article: Strategies and processes for high quality wire arc additive manufacturing. Addit. Manuf..

[8] Mehnen J., Ding J., Lockett H., Kazanas P. (2014). Design study for wire and arc additive manufacture. Int. J. Prod. Dev..

[9] Bermingham M.J., Nicastro L., Kent D., Chen Y., Dargusch M.S. (2018). Optimising the mechanical properties of Ti-6Al-4V components produced by wire+ arc additive manufacturing with post-process heat treatments. J. Alloys Compd..

[10] Asala G., Khan A.K., Andersson J., Ojo O.A. (2017). Microstructural analyses of ATI 718Plus^®^ produced by wire-ARC additive manufacturing process. Metall. Mater. Trans. A.

[11] Dhinakaran V., Ajith J., Fahmidha A.F.Y., Jagadeesha T., Sathish T., Stalin B. (2020). Wire Arc Additive Manufacturing (WAAM) process of nickel based superalloys—A review. Mater. Today Proc..

[12] Lu X., Zhou Y.F., Xing X.L., Shao L.Y., Yang Q.X., Gao S.Y. (2017). Open-source wire and arc additive manufacturing system: Formability, microstructures, and mechanical properties. Int. J. Adv. Manuf. Technol..

[13] Feucht T., Lange J., Waldschmitt B., Schudlich A.K., Klein M., Oechsner M. (2020). Welding Process for the Additive Manufacturing of Cantilevered Components with the WAAM. Advanced Joining Processes.

[14] Ghaffari M., Nemani A.V., Rafieazad M., Nasiri A. (2019). Effect of solidification defects and HAZ softening on the anisotropic mechanical properties of a wire arc additive-manufactured low-carbon low-alloy steel part. JOM.

[15] Müller J., Grabowski M., Müller C., Hensel J., Unglaub J., Thiele K., Dilger K. (2019). Design and Parameter Identification of Wire and Arc Additively Manufactured (WAAM) Steel Bars for Use in Construction. Metals.

[16] Rafieazad M., Ghaffari M., Nemani A.V., Nasiri A. (2019). Microstructural evolution and mechanical properties of a low-carbon low-alloy steel produced by wire arc additive manufacturing. Int. J. Adv. Manuf. Technol..

[17] Gordon J., Hochhalter J., Haden C., Harlow D.G. (2019). Enhancement in fatigue performance of metastable austenitic stainless steel through directed energy deposition additive manufacturing. Mater. Des..

[18] Wu W., Xue J., Wang L., Zhang Z., Hu Y., Dong C. (2019). Forming process, microstructure, and mechanical properties of thin-walled 316L stainless steel using speed-cold-welding additive manufacturing. Metals.

[19] Ali Y., Henckell P., Hildebrand J., Reimann J., Bergmann J.P., Barnikol-Oettler S. (2019). Wire arc additive manufacturing of hot work tool steel with CMT process. J. Mater. Process. Technol..

[20] Chen X., Su C., Wang Y., Siddiquee A.N., Sergey K., Jayalakshmi S., Singh R.A. (2018). Cold metal transfer (CMT) based wire and arc additive manufacture (WAAM) system. J. Surf. Invest..

[21] Da Silva L.J., Souza D.M., de Araújo D.B., Reis R.P., Scotti A. (2020). Concept and validation of an active cooling technique to mitigate heat accumulation in WAAM. Int. J. Adv. Manuf. Technol..

[22] Shi J., Li F., Chen S., Zhao Y., Tian H. (2019). Effect of in-process active cooling on forming quality and efficiency of tandem GMAW–based additive manufacturing. Int. J. Adv. Manuf. Technol..

[23] Fritz A.H. (2018). neu bearbeitete und ergänzte Auflage. Fertigungstechnik. 12.

[24] Schwartz M.M., Olson D.L., Siewert T.A., Liu S., Edwards G.R. (1993). Welding, Brazing and Soldering. ASM Handbook.

[25] Trube S., Miklos E. (2001). Metall-Aktivgasschweißen MAG/Metall-Inertgasschweißen MIG. Proceedings of 29.

[26] Nadzam J., Armao F., Byall L., Kotecki D., Miller D. (2014). Gas Metal Arc Welding—Product and Procedure Selection.

[27] A Green Perspective on Arc Welding Part 1: Harnessing the Power of Resistive Heating. https://ewi.org/a-green-perspective-on-arc-welding-part-1-harnessing-the-power-of-resistive-heating/.

[28] Zhan Q., Liang Y., Ding J., Williams S. (2017). A Wire Deflection Detection Method Based on Image Processing in Wire + Arc Additive Manufacturing. J. Adv. Manuf. Technol..

[29] Dilthey U. (2006). Schweiß- und Schneidtechnologien, 3. bearbeitete Auflage In Schweißtechnische Fertigungsverfahren 1.

[30] Weman K., Lindén G. (2006). MIG Welding Guide.

[31] Barhorst S., Cary H. Synergic Pulsed GMAW In Perspective. Proceedings of the International Conference on Welding for Challenging Enviroments.

[32] Siewert E. (2013). Experimentelle Analyse des Elektrodenwerkstoffübergangs beim Metallschutzgasschweißen mit gepulstem Schweißstrom. Ph.D. Thesis.

[33] Richter F. (2010). The Physical Properties of Steels “The 100 Steels Programme” Part I: Tables and Figures. Ph.D. Thesis.

[34] Matthes K.J., Schneider W. (2016). Schweißtechnik, Schweißen von metallischen Konstruktionswerkstoffen.

[35] (2018). DIN EN ISO 6507-1:2018-07: Metallic Materials—Vickers Hardness Test—Part 1: Test Method (ISO 6507-1:2018).

[36] (2001). DIN EN 1011-2:2001-05: Welding—Recommendation for Welding of Metallic Materials—Part 2: Arc Welding of Ferritic Steels.

[37] Seyffarth P., Meyer B., Scharff A. (2018). Großer Atlas Schweiß-ZTU-Schaubilder.

